# Understanding Inhomogeneous Reactions in Li‐Ion Batteries: Operando Synchrotron X‐Ray Diffraction on Two‐Layer Electrodes

**DOI:** 10.1002/advs.201500083

**Published:** 2015-05-22

**Authors:** Tsuyoshi Sasaki, Claire Villevieille, Yoji Takeuchi, Petr Novák

**Affiliations:** ^1^ Battery LaboratoryToyota Central R&D Labs, Inc.41‐1 YokomichiNagakuteAichi480‐1192Japan; ^2^ Electrochemical Energy Storage SectionPaul Scherrer InstituteCH‐5232Villigen PSISwitzerland

**Keywords:** electrochemistry, homogeneity, in situ X‐ray diffraction, lithium‐ion batteries

## Abstract

To understand inhomogeneous reactions perpendicular to the current collector in an electrode for batteries, a method combining operando synchrotron X‐ray diffraction and two‐layer electrodes with different porosities is developed. The two layers are built using two different active materials (LiNi_0.80_Co_0.15_Al_0.05_O_2_ and LiMn_2_O_4_), therefore, tracing each diffraction pattern reveals which active material is reacting during the electrochemical measurement in transmission mode. The results demonstrate that the active material close to the separator is obviously more active than that one close to the current collector in the case of low porosity electrodes. This inhomogeneity should be due to the rate‐limitation and especially to low average ionic conductivity of the electrolyte in the porous electrode because the current flows first mainly into the electrode regions close to the separator. The inhomogeneity is found to be mitigated by the adjustment of the electrode density and thus porosity. Hence, the novel operando method reveals a clear inhomogeneous reaction perpendicular to the current collector.

## 1 Introduction

Over the past decades, a considerable number of studies have been conducted on the durability and safety of lithium‐ion batteries. The remaining challenge related to these issues is the fact that there are some significant inhomogeneous reactions in practical Li‐ion batteries.^1^, ^2^, ^3^ An inhomogeneous reaction leads to a concentration of current flow into a part of the electrode which then suffers from overuse and overcharge behavior. The local overuse and overcharge in lithium‐ion batteries makes the lifetime shorter and the safety lower, respectively. In an electrode there are two types of inhomogeneities, in lateral direction and in perpendicular direction. Several studies have been conducted on the lithium distribution in the lateral direction in electrodes.^1^, ^2^, ^3^, ^4^, ^5^, ^6^ However, only a few studies have been made regarding the inhomogeneous lithium distribution in the direction perpendicular to the current collector.^7^, ^8^ Furthermore, to understand the inhomogeneous reactions, operando measurements are the primary goal – to follow the lithium distribution in real time and to avoid relaxation problems coming from ex situ experiments.

The objective of this work was thus to develop a powerful in situ method to investigate the inhomogeneous reactions in the perpendicular direction. In situ synchrotron X‐ray diffraction (XRD) measurements offer one of the most powerful and nondestructive tools to explore the state of charge (SOC) and the lithium content of active materials in Li‐ion cells by detecting structural changes.^9^, ^10^ Synchrotron's source is required for this kind of study because it allows very fast and successive measurements especially in transmission mode. To detect inhomogeneous reactions, we fabricated “two‐layer” electrodes as model electrodes for the detection of the inhomogeneities and performed in situ XRD measurements in transmission mode. In this study, two materials, LiNi_0.80_Co_0.15_Al_0.05_O_2_ and LiMn_2_O_4_, are used in the two‐layer electrodes for the in situ XRD measurements. For the analyses of the rate‐limitation factor of the inhomogeneous reactions, the dependency on the porosity (density) of the electrodes was also investigated. Generally, the inhomogeneous reactions in the lateral and perpendicular directions will occur simultaneously. To minimize the effect of the inhomogeneity in the lateral direction, we made the comparison study of the two‐layer electrodes with the different order of layers or the porosity in the same configuration.

## 2 Results and Discussion

**Figure 1:**
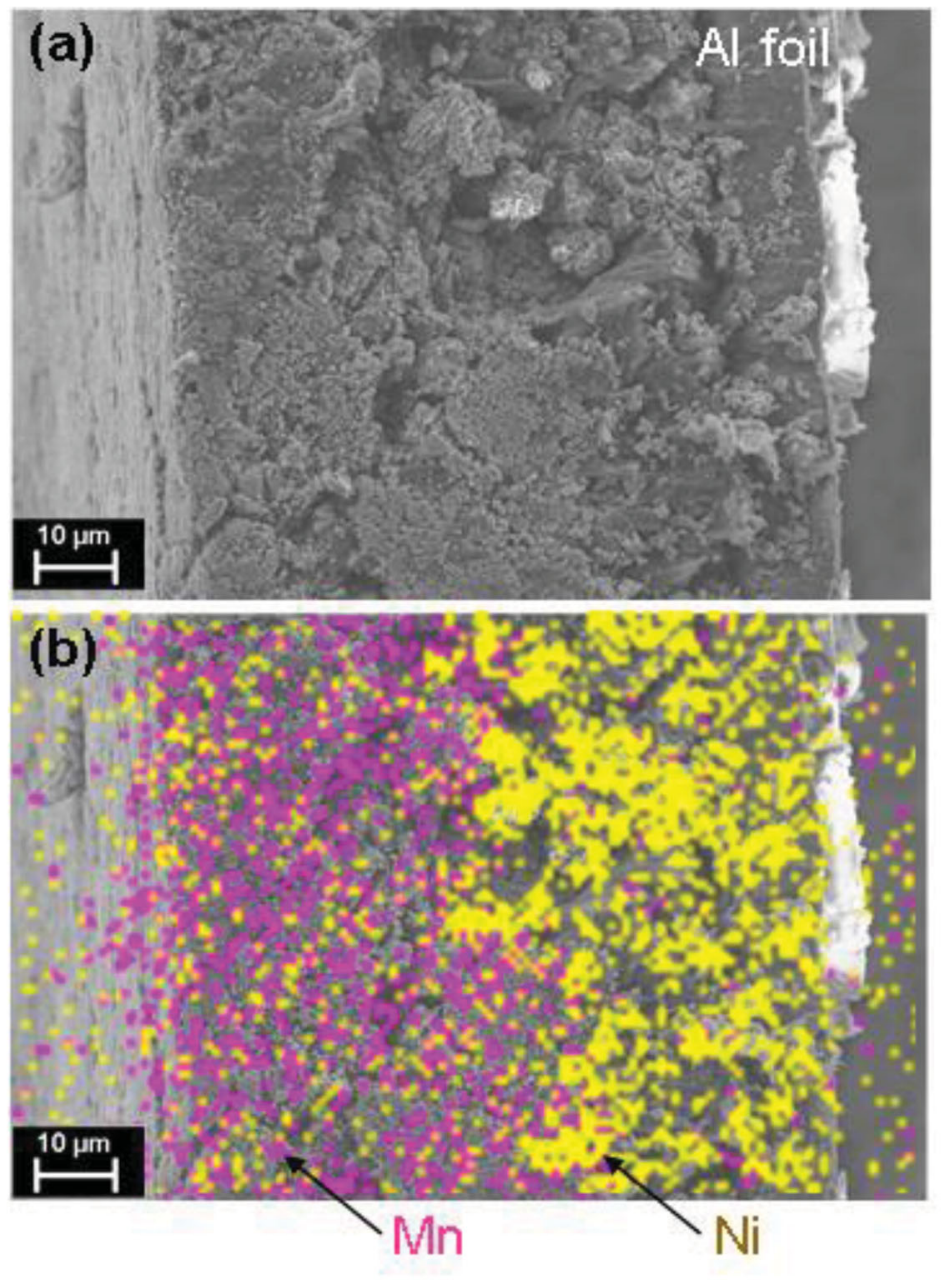
a) SEM image and b) EDX mapping of a two‐layer electrode. The colored dots represent the elemental map for Mn (pink) and Ni (yellow).

**Figure** **1**a shows the cross‐section image of the typical two‐layer electrode recorded by scanning electron microscopy (SEM). The two‐layer structure is clearly visible in Figure 1b, from a mapping with energy dispersive X‐ray spectroscopy (EDX). The result of EDX mapping clearly confirms the two‐layer structure of LiNi_0.80_Co_0.15_Al_0.05_O_2_ (NCA) and LiMn_2_O_4_ (LMO) based electrode. In order to have a fair comparison, four kinds of two‐layer electrodes were made using different order of layers or different electrode density (which correlates with the average electrode porosity). The mass of every active material is almost same in all two‐layer electrodes. Sample H1 has LMO on the separator (upper) side and NCA on the current collector (lower) side with high packing density (2.9 g cm^−3^, lower porosity (≈16%)), and vice versa for the sample H2. Samples L1 and L2 have the same layer configuration as H1 and H2 with low packing density (2.4 g cm^−3^, higher porosity (≈31%)), respectively.

**Figure 2:**
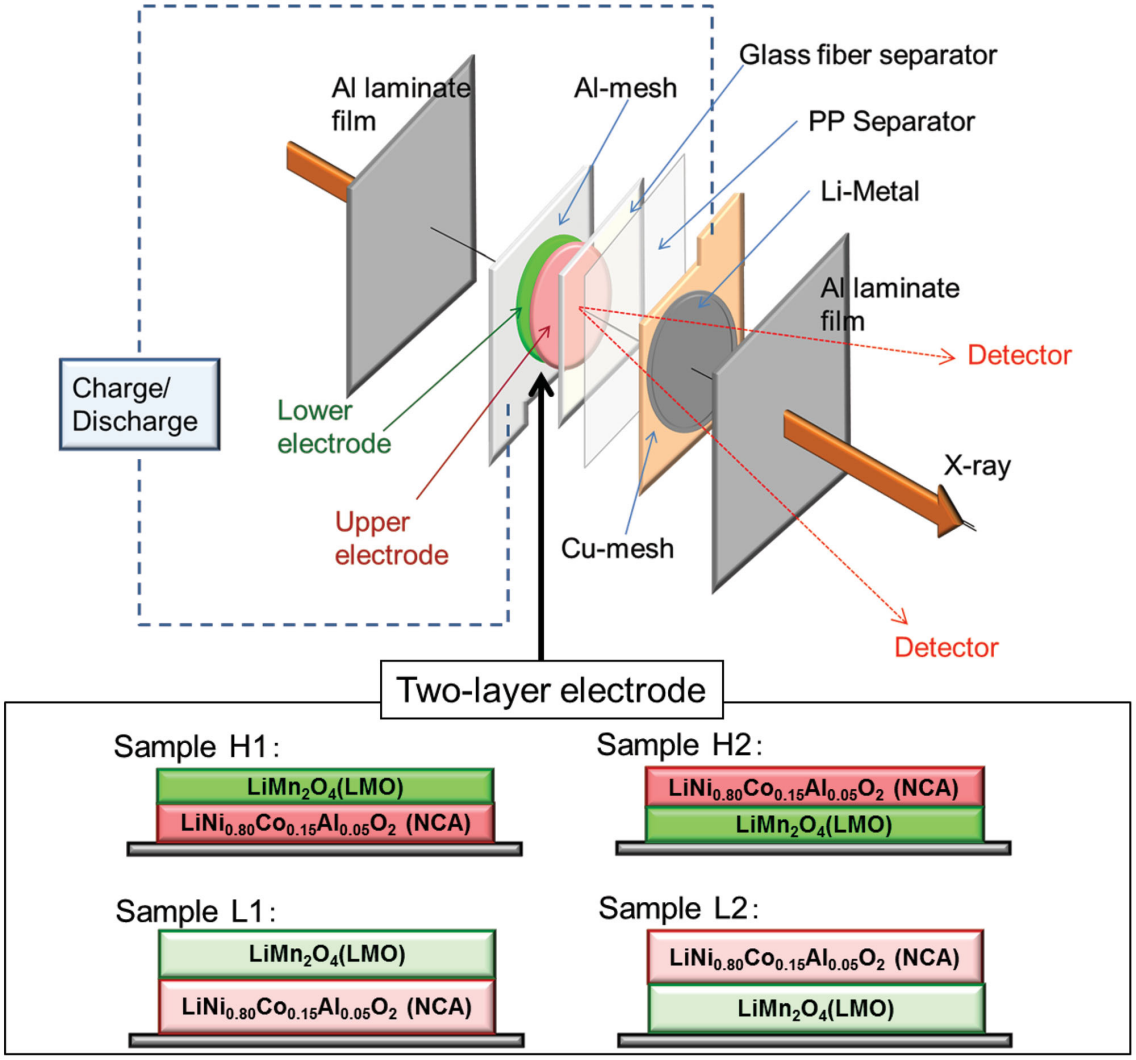
Schematic view of the “coffee‐bag” cell on the synchrotron X‐ray beam (top) and the two‐layer electrode configurations (bottom).

For the in situ X‐ray diffraction measurements, “coffee‐bag” cells were used.^11^ In **Figure** **2**, a schematic drawing of the “coffee‐bag” cell on the X‐ray beamline is shown as well as the configuration of the four types of two‐layer electrodes.

**Figure 3:**
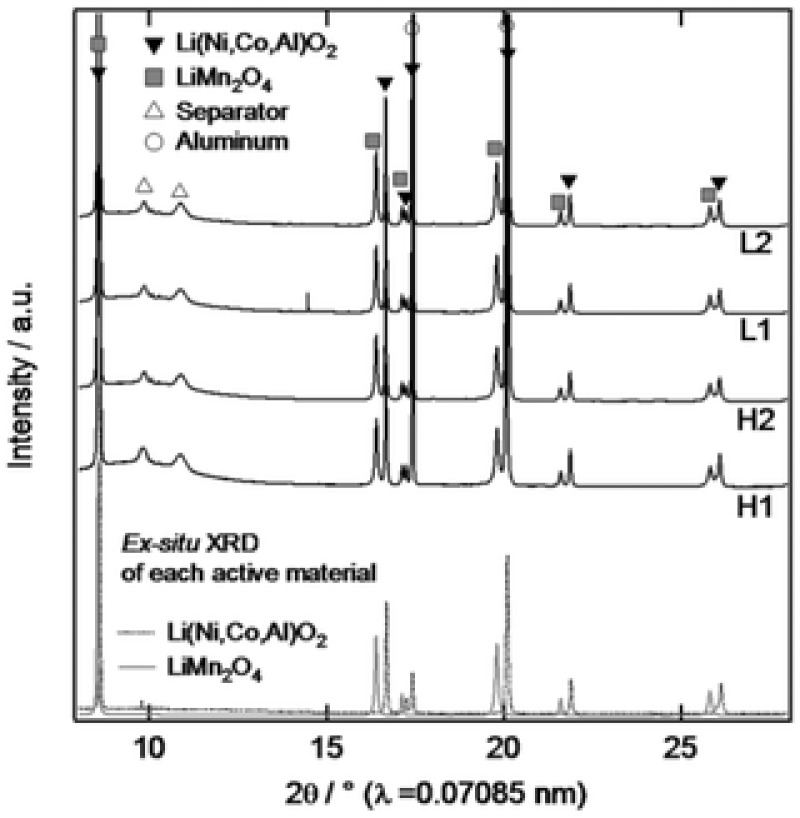
XRD patterns of the samples H1, H2, L1, and L2 inside the “coffee‐bag” cells at open circuit voltage before the electrochemical measurements and ex situ XRD patterns of LMO and NCA.

**Figure 4:**
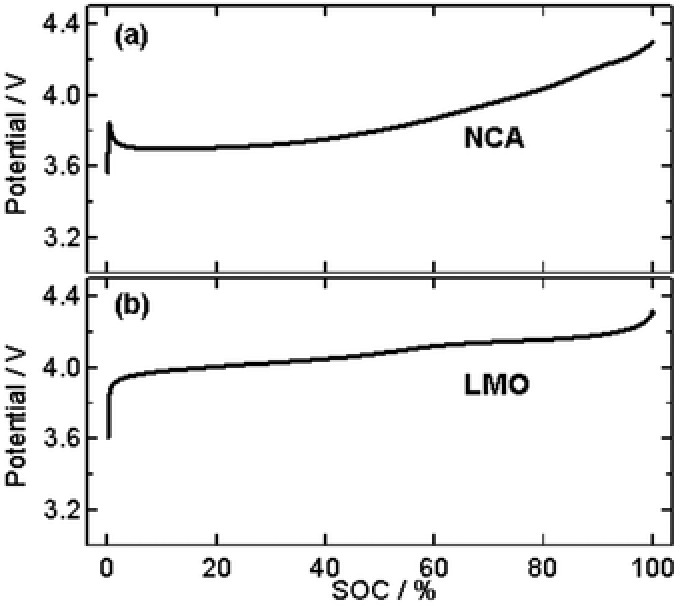
Galvanostatic charge/discharge curves of LMO and NCA.

**Figure 5:**
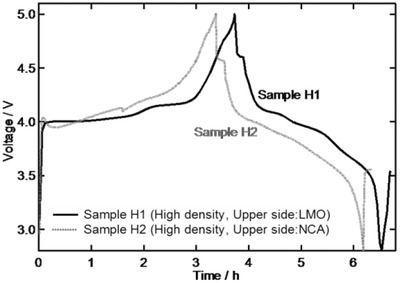
Galvanostatic charge/discharge curves of the samples H1 and H2 at C/4 rate recorded during the in situ XRD measurements.

**Figure** **3** shows the XRD patterns of four samples, H1, H2, L1, and L2, inside the “coffee‐bag” cells at open circuit voltage before the electrochemical measurements together with the ex situ XRD patterns of LMO and NCA. The two‐layer electrodes show identical XRD patterns matching with LMO and NCA indexation. For reference, the electrochemical behavior of each active material was confirmed in standard two‐electrodes coin‐like cells.^11^ **Figure** **4** shows the first charge curves of LMO and NCA at C/4 rate. Note that the NCA showed a characteristic overshoot at the beginning of the first charge curve. **Figure** **5** shows the first charge and discharge curves of the “coffee‐bag” cells with the high‐density samples, H1 and H2, operated in voltage window from 2.8 to 5.0 V at C/4 rate during the in situ XRD measurements.

The two‐layer electrodes showed higher polarizations because the thickness of the electrodes was double compared with single‐layer electrodes as shown in Figure 4. As shown in Figure 3, the starting X‐ray diffraction patterns are similar; however, the electrochemical behavior was different depending on the order of the two‐layer configuration. Sample H1 showed a flat region in the beginning of the charge curve like LMO, as shown in Figure 4b. On the other hand, sample H2 showed a characteristic overshoot at the beginning of the charge curve like NCA, as shown in Figure 4a. The charge curves thus already indicate a significant difference, preferring the reaction of the phase close to the separator. Such behavior is directly related to inhomogeneous reactions in the electrodes in the direction perpendicular to the current collector. Due to the higher binder contents than typical electrodes in commercial Li‐ion batteries, the inhomogeneity of reactions in these two‐layer electrodes might be exaggerated. However, these two‐layer electrodes were used as model electrodes to develop a novel method to detect the inhomogeneous reactions.

**Figure 6:**
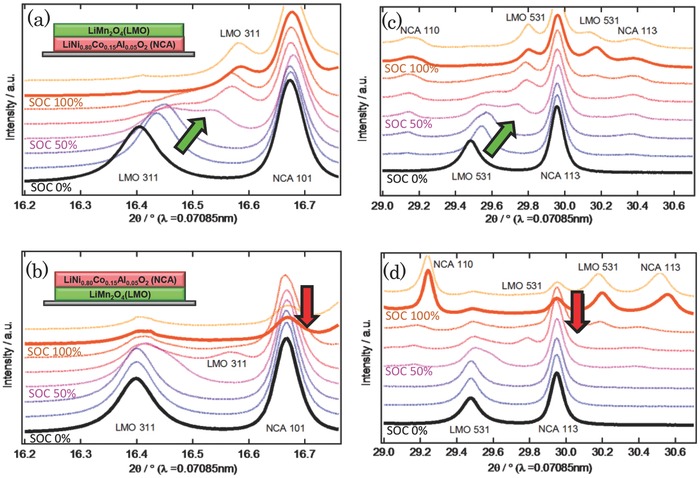
In situ XRD patterns collected during the first charge up to 5.0 V; a) sample H1 and b) sample H2 in 2*θ* = 16.2–16.8°, c) sample H1 and d) sample H2 in 2*θ* = 29.0–30.7°.

**Figure** **6** shows in situ XRD patterns of samples H1 and H2 collected during the first charge up to 5.0 V. In the diffraction angle range 2*θ* = 16.2–16.8° and 29.0–30.7°, one peak from LMO and one peak from NCA can be detected in the initial stage, respectively. The 101 and 113 reflection peaks of NCA show two‐phase like reaction during charge/discharge process, therefore, the peak intensities change during charge process as shown in the Supporting Information. The changes in the peak intensities and/or positions are good indicators to detect which active material is reacting. If the reaction is homogeneous, the change of the in situ diffraction pattern should be identical for both samples but actually such a behavior is NOT observed. Analyzing the in situ XRD patterns change of samples H1 (Figure 6a) and H2 (Figure 6b), the peak of LMO in sample H1 showed a faster shift than that in sample H2 (red arrow in Figure 6a). On the other hand, the in situ XRD pattern change of the NCA peak for sample H2 showed a faster intensity dropping than that for sample H1 (green arrow in Figure 6b). The peak shifts or the intensity dropping indicate the respective reaction of the active materials, therefore, the active material close to the separator side was obviously more active (i.e., reacted faster) than that close to the current collector side. In other words, the current in the electrodes tended to “rush” into the separator side in the case of the high‐density (lower porosity) electrodes. This phenomenon seems to be strange from the point of view of the redox potential of the two active materials. As shown in Figure 4, the redox potential of NCA is lower than that of LMO, therefore the NCA should be responsible for the charging reaction at the early stage. The behavior of H1 sample seems to be abnormal. To explain this phenomenon, we should take into account the overall kinetics, i.e., the inhomogeneous reaction in the perpendicular direction to the current collector.

This inhomogeneity of high‐density electrodes is probably due to the rate‐limitation as a consequence of the rather low ionic conductivity of the electrolyte phase in the porous electrode. Thus, the active material at the separator side will “see” a lower total ionic resistance than that at the current collector side. This behavior of the inhomogeneous reactions as shown in Figure 6 is consistent with the electrochemical signature of the two‐layer electrodes (Figure 5). In sample H1, the LMO layer shows a faster reaction than the NCA layer. This is the reason why the charge curve of sample H1 in Figure 5 showed the flat region in the beginning like LMO and vice versa for the sample H2.

**Figure 7:**
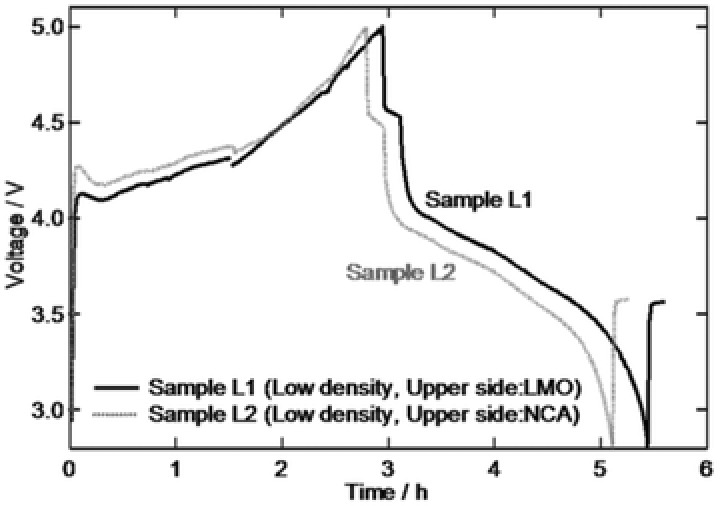
Galvanostatic charge/discharge curves of the samples L1 and L2 at C/4 rate recorded during the in situ XRD measurements.

To confirm the rate‐limitation effect of the ionic conductivity, the comparison between low‐ and high‐density electrodes is valuable. **Figure** **7** shows the first charge and discharge curves of the “coffee‐bag” cells with the low‐density samples, L1 and L2, during the in situ XRD measurements. Sample L1 has the same layer order as sample H1, however, sample L1 showed no flat region in the beginning like LMO and sample H1.

**Figure 8:**
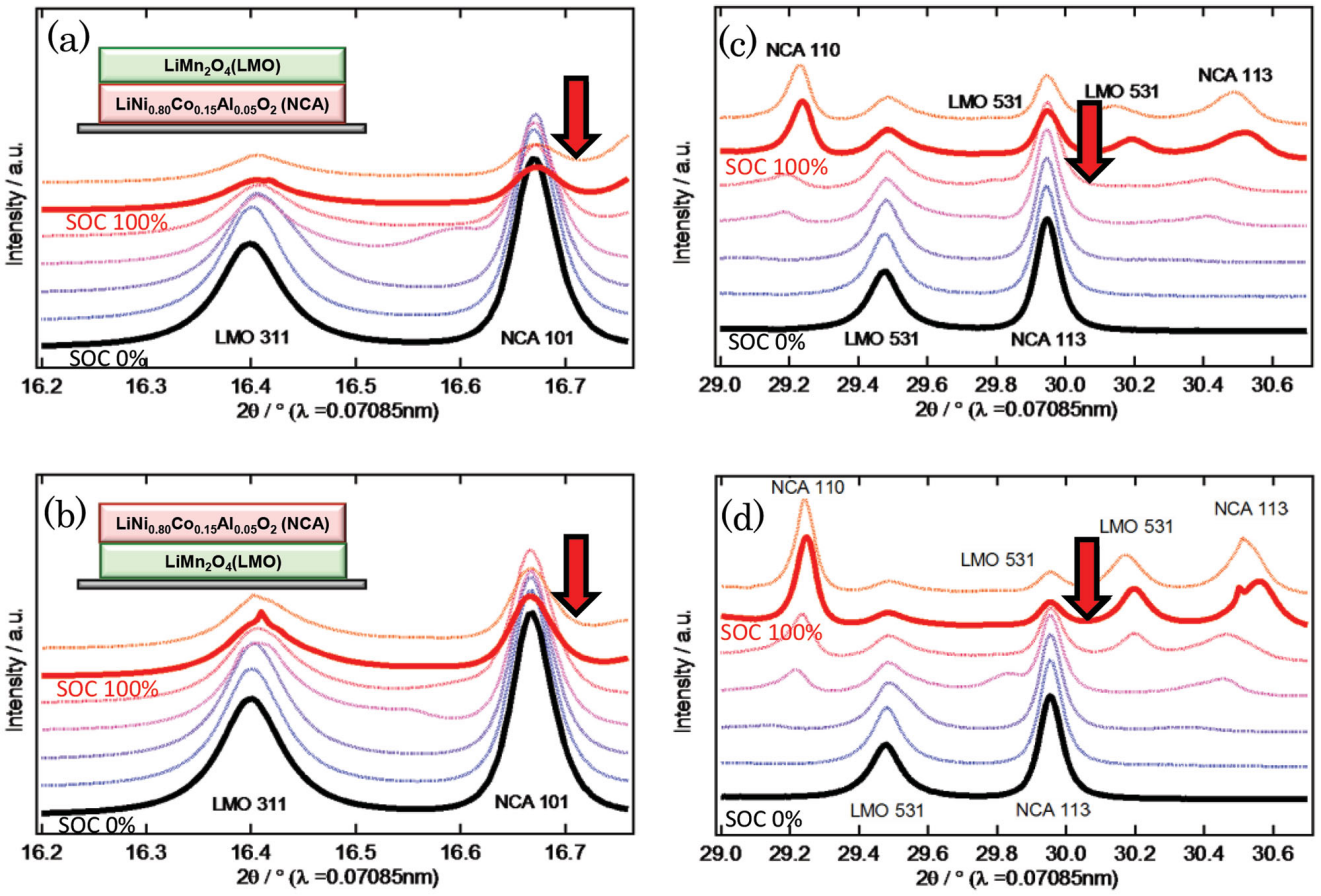
In situ XRD patterns collected during the first charge up to 5.0 V; a) sample L1 and b) sample L2 in 2*θ* = 16.2–16.8°, c) sample L1 and d) sample L2 in 2*θ* = 29.0–30.7°.

**Figure** **8** shows in situ XRD patterns of samples L1 and L2 collected during the first charge up to 5.0 V. In contrast with sample H1, sample L1 showed that NCA, the active material closer to the current collector side, was more active. This means that the ionic conductivity of the low‐density (higher porosity) electrodes was NOT rate‐limiting. Additionally, the in situ XRD patterns of samples L1 and L2 showed little differences; therefore we can conclude that the low‐density electrodes showed less inhomogeneity than the high density electrodes in this particular case. In other words, the current distribution perpendicular to the current collector can be controlled by the adjustment of the electrode density (i.e., the electrode porosity). The effects of temperature, thickness, and charge/discharge current are also important factor to change the degree of the inhomogeneities as shown in the other references.^4^, ^7^ On the other hand, the inhomogeneous reaction in the perpendicular direction is due to the unbalance between the ionic and electronic conductivities, therefore, the porosity can change the origin, the ionic and electronic conductivities directly. For example, lower porosity will lead to lower ionic conductivity and higher electronic conductivity, such as H1 or H2 electrodes. Thus, the porosity is supposed to be one of the main factors of the inhomogeneous reactions as well as temperature, thickness, and charge/discharge current.

## 3 Conclusion

These studies of the inhomogeneous reactions offer a starting point for improving the electrode's stability against overcharge and overuse. To understand the durability and safety of Li‐ion batteries, detailed investigations of inhomogeneous reaction during in situ/operando conditions are crucial. The inhomogeneous reactions perpendicular to the current collector will depend on many factors such as (i) configuration of the electrodes, (ii) porosity, (iii) thickness, (iv) amount of conductive carbon, (v) current rate, and so on. Our novel in situ method revealed clearly the capability to detect inhomogeneous reactions perpendicular to the current collector. We are convinced that this study can pave a new path for in situ/operando investigations of inhomogeneous processes not only in electrodes but in many potential applications.

## 4 Experimental Section

*“Two‐Layer” Electrode*: The two‐layer electrodes were built of two self‐standing films with different active materials, NCA and LMO. The self‐standing films were prepared by mixing 80 wt% of the respective active material, 5 wt% of conductive carbon (Imerys SuperP), and 15 wt% of PVdF‐Hexafluoropropylene (HFP) copolymer binder (Kynar Flex 2801). The two self‐standing films were superimposed and then calendared together at 80 °C onto an Al foil (serving as current collector) into the appropriate packing density (2.9 g cm^−3^ or 2.4 g cm^−3^). The physical properties of the two‐layer electrodes were summarized in **Table** **1**. The estimated porosities were the average values of the whole electrodes. The porosities of NCA and LMO could not be controlled independently. However, for example, each NCA (or LMO) in high‐density electrodes, sample H1 and H2, should have the same porosities, because the calendaring process and the controlled thicknesses were same as shown in **Table** **2**. The key point of this approach was the comparison with the different orders of layer in the same configurations; thicknesses, each loading weights, and porosities. It allowed to make fair comparisons of the effect of the inhomogeneous reactions perpendicular to the current collector.

**Table 1:** Physical properties of two‐layer electrodes with different densities and order of the layers

Sample	Density [g cm^−3^]	Thickness [μm]	Loading weight [mg cm^−2^]	Porosity [%]
H1	2.89	83	24.0	15.5
H2	2.89	85	24.5	15.7
L1	2.36	99	23.4	31.1
L2	2.38	98	23.3	30.6

**Table 2:** Performances of the MYTHEN detector system

Intrinsic angular resolution	0.04
Strip pitch	50 mm
Strip length	8 mm
Number of channels	15 360
Readout time	250 ms

The molar ratio of NCA to LMO was between 0.93 and 0.97. The assembled electrodes were used as working electrodes for the in situ measurements. The structural changes of NCA and LMO during the charge/discharge process were reported by our group^12^ and others.^9^, ^10^ Thus it was detected that which active material was reacting during the in situ measurement by tracing each XRD pattern.

*Electrochemical In Situ Measurement*: For the in situ XRD measurements, “coffee‐bag” cells were used^11^ as shown in Figure 2. Li metal was used as a counter electrode, and a 25 μm monolayer polypropylene (PP) separator (Celgard 2400, Celgard, USA) and a glass fibers separator (GF/G3 grade, Whatman, UK) were soaked in 1 m LiPF_6_ in ethylene carbonate (EC)/dimethyl carbonate (DMC) electrolyte. The glass fibers separator was needed as an electrolyte absorber. The assembled “coffee‐bag” cells were loaded into an automatic sample changer.^13^ Then, in situ XRD patterns were collected at the MS‐powder beamline (X04SA) at Swiss Light Source (SLS) at the Paul Scherrer Institut (PSI) at room temperature. The XRD patterns were collected in transmission mode using a 17.5 keV X‐ray beam. The beam spot size was 0.5 × 0.5 mm. A MYTHEN (Microstrip sYstem for Time‐rEsolved experimeNts) detector system was used to collect the XRD patterns in the range of −60° to −5° and 5° to 60° in 2*θ*.^14^ The detector system characteristics were summarized in Table 1.^13^, ^14^

The FORTRAN program postpro_AC_0.3d which developed at SLS performed the processing of the raw data from the MYTHEN detector.^15^ The routine included (1) conversion of detector channels to diffraction angles, (2) merging of datasets collected at different detector positions, (3) a flat‐field correction, (4) the elimination of dead or hot detector channels, and (5) a normalization to the data to reflect any changes in the incident X‐ray beam intensity.^15^ The electrochemical analyses were carried out galvanostatically at C/4 rate between 2.8 and 5.0 V versus Li^+^/Li.

The measurement time of each in situ XRD pattern was 4 min. However, the interval time for each measurement was about 30 min due to the sequential multimeasurements of several cells by using the automatic sample changer.^13^ At least seven or eight data points were needed to trace the reaction during the charge/discharge process. This was the reason for using C/4 rate to record the galvanostatic charge/discharge curves of the samples.

This experimental configuration with the “coffee‐bag” cells and thinner electrodes showed good correlations between electrochemical behavior and in situ XRD patterns during charge/discharge between 3.0 and 5.1 V without any significant delay due to the inhomogeneous reactions in the lateral direction.^12^ This configuration provided good conditions to investigate inhomogeneous reactions perpendicular to the current collector.

## Supporting information

As a service to our authors and readers, this journal provides supporting information supplied by the authors. Such materials are peer reviewed and may be re‐organized for online delivery, but are not copy‐edited or typeset. Technical support issues arising from supporting information (other than missing files) should be addressed to the authors.

Supplementary
