# Supplementary material for: Understanding Inhomogeneous Reactions in Li‐Ion Batteries: Operando Synchrotron X‐Ray Diffraction on Two‐Layer Electrodes
Source: Adv Sci (Weinh). 2015 May 22;2(7):1500083. doi: 10.1002/advs.201500083 (PMC5033016; doi:10.1002/advs.201500083)
Supplement: Supplementary file 1 — Supplementary [file ADVS-2-0l-s001.pdf]

Copyright WILEY-VCH Verlag GmbH & Co. KGaA, 69469 Weinheim, Germany, 2015.

ADVANCED SCIENCE
Open Access

Supporting Information

for *Adv. Sci.*, DOI: 10.1002/advs.201500083

Understanding Inhomogeneous Reactions in Li-Ion Batteries: Operando Synchrotron X-Ray Diffraction on Two-Layer Electrodes

Tsuyoshi Sasaki, Claire Villevieille, Yoji Takeuchi, and Petr Novák **

Supplementary Information S1:

In-situ XRD patterns for a single-layer electrode of NCA

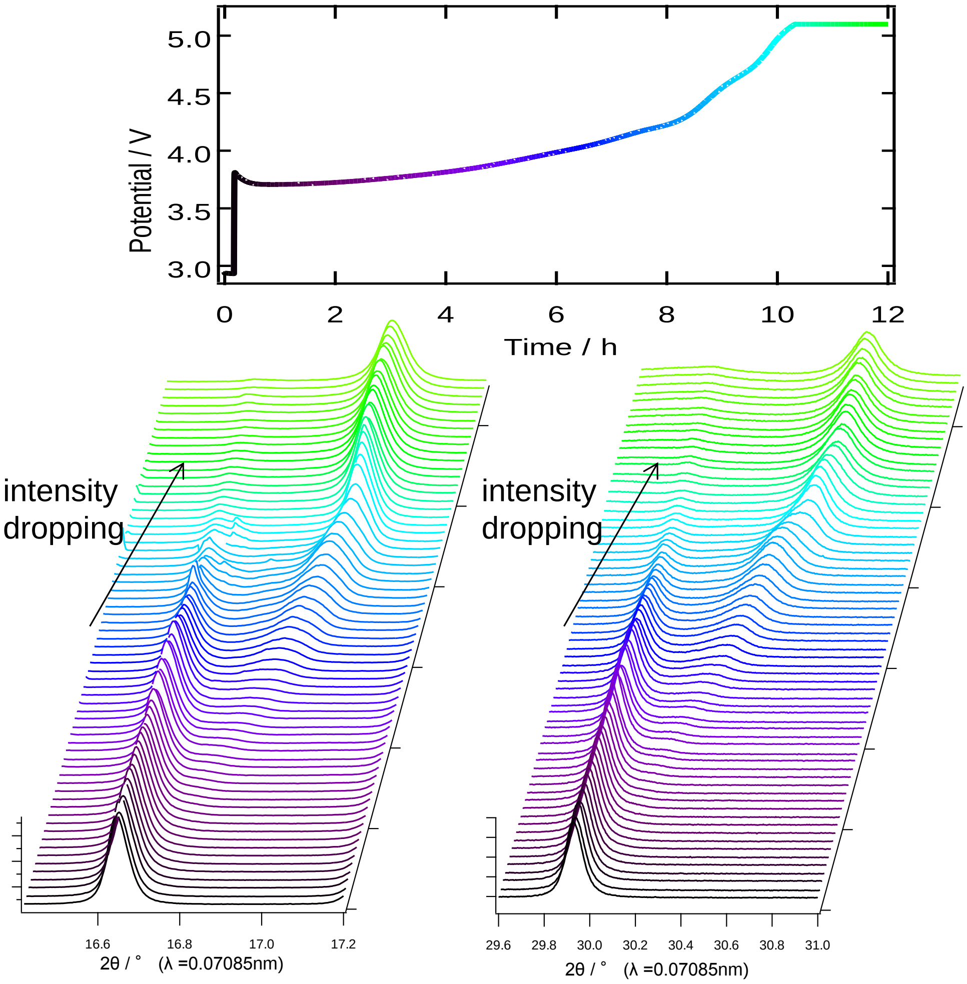

Figure S1. a) The charge curve of "coffee-bag cell" with a NCA single-layer electrode between 3.0 and 5.1 V at a C/10 rate and b) *in situ* XRD patterns in the 101 and 113 reflection region (2θ = 16.4-17.2° and 29.6-31.0°) collected during the charge of the NCA single-layer electrode . The color of each XRD pattern corresponds to the color of the charge curve [12].
